# The burden of cardiovascular diseases in Ethiopia from 1990 to 2017: evidence from the Global Burden of Disease Study

**DOI:** 10.1093/inthealth/ihaa069

**Published:** 2020-09-18

**Authors:** Solomon Ali, Awoke Misganaw, Asnake Worku, Zelalem Destaw, Legesse Negash, Abebe Bekele, Paul S Briant, Catherine O Johnson, Tahiya Alam, Chris Odell, Gregory A Roth, Mohsen Naghavi, Ebba Abate, Alemnesh H Mirkuzie

**Affiliations:** Ethiopian Public Health Institute, Addis Ababa, Ethiopia; Saint Paul’s Hospital Millennium Medical College, Addis Ababa, Ethiopia; Ethiopian Public Health Institute, Addis Ababa, Ethiopia; Institute for Health Metrics and Evaluation, Department of Health Metrics Sciences, University of Washington, Seattle, WA 98121, USA; Ethiopian Public Health Institute, Addis Ababa, Ethiopia; Ethiopian Public Health Institute, Addis Ababa, Ethiopia; Ethiopian Public Health Institute, Addis Ababa, Ethiopia; Ethiopian Public Health Institute, Addis Ababa, Ethiopia; Institute for Health Metrics and Evaluation, Department of Health Metrics Sciences, University of Washington, Seattle, WA 98121, USA; Institute for Health Metrics and Evaluation, Department of Health Metrics Sciences, University of Washington, Seattle, WA 98121, USA; Institute for Health Metrics and Evaluation, Department of Health Metrics Sciences, University of Washington, Seattle, WA 98121, USA; Institute for Health Metrics and Evaluation, Department of Health Metrics Sciences, University of Washington, Seattle, WA 98121, USA; Institute for Health Metrics and Evaluation, Department of Health Metrics Sciences, University of Washington, Seattle, WA 98121, USA; Institute for Health Metrics and Evaluation, Department of Health Metrics Sciences, University of Washington, Seattle, WA 98121, USA; Ethiopian Public Health Institute, Addis Ababa, Ethiopia; Ethiopian Public Health Institute, Addis Ababa, Ethiopia; University of Bergen, Norway

**Keywords:** cardiovascular diseases, EPHI, Ethiopia, mortality

## Abstract

In Ethiopia, evidence on the national burden of cardiovascular diseases (CVDs) is limited. To address this gap, this systematic analysis estimated the burden of CVDs in Ethiopia using the Global Burden of Disease (GBD) 2017 study data. The age-standardized CVD prevalence, disability-adjusted life years (DALYs) and mortality rates in Ethiopia were 5534 (95% uncertainty interval [UI] 5310.09 - 5774.0), 3549.6 (95% UI 3229.0 - 3911.9) and 182.63 (95% UI 165.49 - 203.9) per 100 000 population, respectively. Compared with 1990, the age-standardized CVD prevalence rate in 2017 showed no change. But significant reductions were observed in CVD mortality (54.7%), CVD DALYs (57.7%) and all-cause mortality (53.4%). The top three prevalent CVDs were ischaemic heart disease, rheumatic heart disease and stroke in descending order. The reduction in the mortality rate due to CVDs is slower than for communicable, maternal, neonatal and nutritional disease mortalities. As a result, CVDs are the leading cause of mortality in Ethiopia. These findings urge Ethiopia to consider CVDs as a priority public health problem.

## Introduction

Cardiovascular conditions, which include coronary heart disease, cerebrovascular disease, peripheral vascular disease, heart failure, rheumatic heart disease (RHD), congenital heart disease and cardiomyopathies, are becoming the leading cause of death in the world.^1^ According to the 2017 World Health Organization global estimate, each year 17.9 million people die from cardiovascular diseases (CVDs) and >75% of these deaths occur in low- and middle-income countries (LMICs).^2^

In low-income countries, CVD mortality and morbidity have become the leading cause of disease burden, overtaking the burden due to infectious diseases such as human immunodeficiency virus (HIV)/acquired immunodeficiency syndrome (AIDS), tuberculosis (TB), respiratory infections, diarrhoea and malaria.^3,4^ Despite this change in the disease landscape, communicable diseases still have the biggest portion of health budgets in most sub-Saharan countries.^3^

Ethiopia is one of the countries in the sub-Saharan Africa region making remarkable progress in tackling priority communicable diseases and maternal, newborn, and child health threats. The country has achieved most of the Millennium Development Goals (MDGs), including reducing the mortality of children <5 y of age by two-thirds, lowering maternal mortality by three-quarters and significantly lowering HIV and TB incidence and mortality.^5,6^ In contrast, little effort has been made to address the challenges of CVDs as a disease priority for public health.^7^

Ethiopia has been one of the fastest-growing economies in East Africa for the past 20 y.^8^ Currently the country is undergoing an epidemiologic transition mainly driven by demographic and lifestyle changes. Rapid urbanization has attracted a large population from rural areas to cities.^9^ Urban life naturally promotes a sedentary lifestyle and increases the chance of CVDs. Considering these facts, the burden of CVDs in Ethiopia might be even higher than anticipated.

There is little published evidence about the burden of CVDs in Ethiopia. Two hospital-based studies from eastern Ethiopia in 1963 and central Ethiopia in 1971 reported CVD prevalences of 7.2% and 24%, respectively.^7,10^ A 2012 population-based study from Addis Ababa and another from Amhara in 2004 estimated mortality attributed to CVDs to be 24% and 6.5%, respectively.^11,12^ All of these studies were done in small segments of the population, in a health facility, have small samples size and show a large variation in the estimates. Thus the validity of these studies to show the national picture of the CVD burden in Ethiopia is questionable.

Ethiopia has adopted the Sustainable Development Goals (SDGs) of the United Nations, and the country has put significant effort into meeting the targets set for HIV, TB, malaria and maternal and child health by 2030.^6^ However, the current effort to address SDG goal 3 subtarget 3.4 of reducing premature mortality from non-communicable diseases (NCDs) by one-third is not enough. In 2008 the Ethiopian Federal Ministry of Health (FMoH) recognized the increasing trend of NCDs through small-scale studies and situational analysis.^13^ Based on the findings, in 2010 the FMoH developed a national strategic framework to prevent and control NCDs. Detailed national and subnational strategic action plans based on the strategic framework as well as guidelines for clinical and programmatic management of major NCDs were prepared in 2016.

There is growing evidence from Ethiopia that identifies several risk factors associated with CVDs. From these, hypertension, physical inactivity, smoking, alcohol consumption, age, high body mass index and abdominal obesity contribute significantly.^14,15^

On the other hand, comprehensive evidence on the burden of CVDs in Ethiopia is scarce. The vital statistics registration system is under development and does not capture deaths or causes of death, although registration of births and marriages is improving. Hospital morbidity and mortality record keeping is also substandard, which reflects the severe evidence gap for CVD morbidity and mortality. To this day, there has been no national survey/surveillance done to estimate the burden of CVDs in Ethiopia. Hence this article aims to fill evidence gaps on CVD burden using the 2017 Global Burden of Diseases, Injuries, and Risk Factors Study (GBD) results. This article estimates CVD incidence, prevalence, mortality, disability-adjusted life years (DALYs), years lived with disability (YLD) and years of life lost (YLL) of CVDs and shows trends and the current burden, with the objective of increasing awareness and informing policy on CVDs in Ethiopia.

## Methodology

### Settings

The Ethiopian Public Health Institute (EPHI) is collaborating with the Institute for Health Metrics and Evaluation (IHME) at the University of Washington on the national burden of disease estimation. All of the results presented in this article were analysed using the GBD 2017 Ethiopian data.

### Data processing approach summary

The GBD data allow a comparison of the magnitude of diseases, injuries and risk factors across age groups, sexes, countries, regions and time. The information generated from the GBD platform can be used to monitor health progress, make informed decisions and indicate policy directions for ministries of health and other stakeholders. It also helps in understanding the leading causes of health loss that could potentially be averted.^16^ The IHME provides GBD results in visualization tools, allowing people to interact with the vast amounts of data and the trends identified therein. These unique tools are beneficial when trying to identify specific information for age groups, sexes, causes, risks and comparisons to other regions.^16^

The GBD 2017 classified 195 countries into 7 superregions and 21 regions based on epidemiological similarities and geographical proximities. Subnational estimates were generated for a selected subset of countries, including Ethiopia.^17^^–19^ Independent estimates of population and fertility were calculated for the first time in GBD 2017,^20,21^ along with estimates of all-cause mortality using a similar approach to GBD 2016. The causes of mortality and morbidity are structured using a four-level classification hierarchy based on the International Classification of Diseases (ICD), used for modelling to produce results that are mutually exclusive and collectively exhaustive.^17–19^

In addition to estimates of deaths and disease prevalence, the GBD produces three summary measures of disease burden: YLL, which are calculated from the sum of each death multiplied by the standard life expectancy at each age;^17^ YLD, which are calculated from the prevalence of each disease sequela multiplied by the disability weight; and DALYs, which are calculated as the sum of YLL+YLD by cause. All fatal, non-fatal and risk estimates are generated by age group and sex for each location from 1990 to 2017. Age-standardized rates are calculated using the GBD standard population. Additional details on estimation methodology are available in the GBD 2017 capstone papers.

Cause-specific deaths are divided into three main groups: communicable, maternal, neonatal and nutritional diseases; NCDs; and injuries. Each of these groups is then further subdivided into disease groupings and then into individual causes. Cause-specific mortality was estimated using the Cause of Death Ensemble model (CODEm).^17^ The CODEm produces few plausible combinations of covariates using a covariate selection algorithm. The combinations of covariates are then analysed using mixed effects linear models and spatiotemporal Gaussian process regression models for cause fractions and death rates.^17^ Further discussion about the model specifications are presented in the GBD 2017 causes of death^18^ and mortality^20^ studies.

The GBD 2017 used the Bayesian meta-regression tool DisMod-MR 2.1 to estimate non-fatal health loss. Custom models or disease-specific natural history models were also used to estimate sequelae and causes when DisModMR 2.1 did not capture the complexity of diseases such as HIV/AIDs or if incidence and prevalence needed to be calculated from other data.^17,19^ Each non-fatal sequela was estimated separately and the occurrence of comorbidity for each age group, sex, location and year group was assessed using a microsimulation framework.^17,19^ Further detail about the estimation methodology used by DisMod-MR 2.1 can be found in the GBD 2017 methods publication.^19^

The GBD 2017 assessed a set of behavioural, environmental or occupational and metabolic risks that were organized into five hierarchical levels for a total of 84 risk groups.^22^ The risk factor analysis established a risk–outcome pair from a combination of risk and outcome association based on evidence rules.^22^ The attributable burden for each risk-outcome pair was calculated using a counterfactual approach to determine what the burden would have been had exposure been reduced to a theoretical minimum exposure level. Additional details of this framework and risk factor exposure estimation are available in the GBD 2017 capstone.^22^

## Results

### General overview

**Figure 1.**
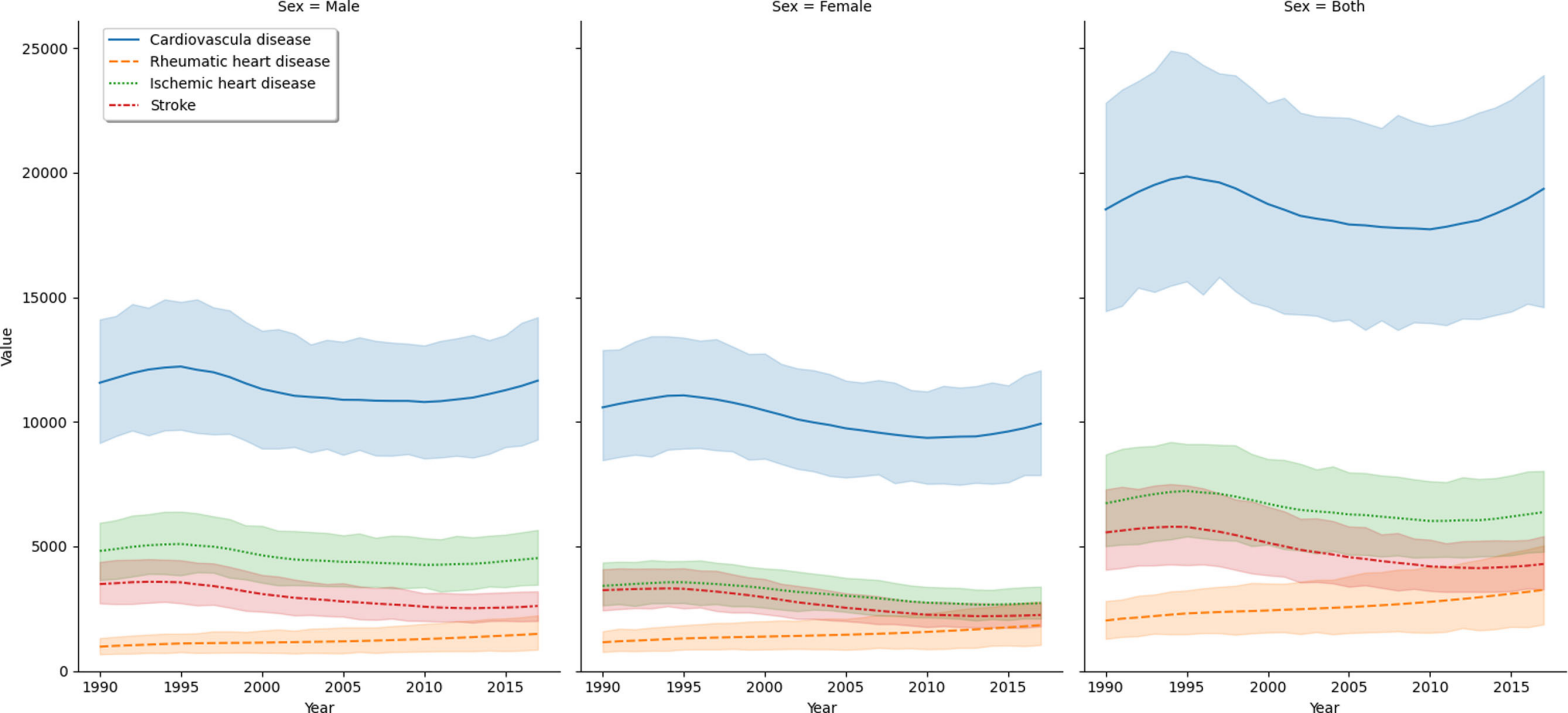
Trends in the age-standardized prevalence rate of the three top cardiovascular diseases from 1990 to 2017, GBD data, Ethiopia. IS: ischaemic stroke; ICH: intracranial haemorrhage; SAH: subarachnoid haemorrhage.

The number of prevalent CVD cases in Ethiopia has shown a 100% increase from 1 398 780 (95% uncertainty interval [UI] 1 343 992 - 1 448 994) in 1990 to 2 838 767 (95% UI 2 725 245 - 2 950 399) in 2017. Fifty-three percent of the cases were males, both in 1990 and in 2017. The age standardized prevalence rate per 100 000 population did not change through time (i.e. 5534 [95% UI 5310.09 - 5774] in 1990 and 5466 [95% UI 5232 - 5695] in 2017). The top three most prevalent CVDs in all ages in 2017 were RHD (prevalence 932.1 [95% UI 886.4 - 979.4]), ischaemic heart disease (IHD; prevalence 621.8 [95% UI 582.3 - 664.9]) and stroke (prevalence 313.8 [95% UI 293.6 - 336.7]). Using the age-standardized metric, IHD prevalence was the leading CVD, followed by RHD and stroke (Figure 1). The all-ages prevalence rate per 100 000 population increased from 2721 (95% UI 2615.0 - 2819.0) in 1990 to 2759 (95% UI 2649.0 - 2868) in 2017, with an annual constant incidence rate and a decline in the death rate from 125.6 (95% UI 108.4 - 143.3) in 1990 to 57.07 (95% UI 51.7 to 65.4) in 2017.

Between 1990 and 2017, the three most prevalent CVDs were RHD, IHD and stroke. The number of prevalent RHD cases has shown a significant increase from 443 184 (95% UI 421 462 - 465 804) in 1990 to 958 956 (95% UI 911 941 - 1 007 628) in 2017. Likewise, the number of prevalent IHD cases has shown a significant increase from 349 512 (95% UI 327 109 - 373 735) in 1990 to 639 466 (95% UI 599 113 - 684) in 2017. The number of prevalent stroke cases has shown a significant increase from 182 662 (95% UI 172 095 - 193 843) in 1990 to 322 818 (95% UI 302 060 - 346 355) in 2017.

In 2017, the age-standardized prevalence rate of RHD for females (1157.86 [95% UI 1101.18 - 1219.59]) was significantly higher than for males (908.56 [95% UI 864.07 to 954.93]). The age-standardized prevalence rate of IHD was significantly higher for males (2047.1 [95% UI 1910.19 - 2192.78]) than for females (1037.84 [95% UI 958.32 - 1119.56]).

### Mortality

Of all CVDs, deaths due to RHD declined by >50% in 2017 compared with 1990. Deaths due to hypertensive heart disease (HHD) and non-rheumatic valvular disease (NRVD) increased in 2017 by 3.1% and 25.6%, respectively, although the increments were not statistically significant (Table 1).

**Table 1.** Total number of CVD deaths, age-standardized mortality rates and percentage of change in Ethiopia from 1990 to 2017, GBD 2017 data

	Deaths					Age-standardized death rate per 100 000				
	1990		2017			1990		2017		
Disease	n	95% UI	n	95% UI	Change, %	Rate	95% UI	Rate	95% UI	Change, %
CVD	64 565	55 722 to 73 663	58 719	53 177 to 65 228	−9.1	402.75	361.2 to 443.37	182.63	165.49 to 203.91	−54.7
RHD	2140	1573 to 2854	1013	728 to 1304	-52.7	8.51	6.65 to 11.25	2.23	1.58 to 2.92	−73.8
IHD	26 853	22 669 to 31 182	26 166	22 169 to 30 366	−2.6	171.66	149.26 to 193.91	82.6	69.39 to 96.52	−51.9
Stroke (overall)	24 592	20 623 to 29 788	19 837	16 841 to 23 139	−19.3	155.26	133.65 to 180.32	62.39	52.44 to 73.51	−59.8
IS	6024	4665 to 7627	6012	4108 to 8039	−0.21	50.51	39.32 to 62.91	22.23	15.28 to 29.36	−56.0
ICH	17 046	13 449 to 21 996	12 611	10 623 to 14 771	−26.0	33.16	26.16 to 42.79	12.26	10.32 to 14.36	−63.0
SAH	1521	843 to 2295	1214	661 to 2450	−20.2	6.95	3.82 to 11.22	2.96	1.51 to 6.27	−57.4
HHD	5718	2718 to 8577	5893	3163 to 9283	3.1	41.56	22.36 to 63.3	19.86	10.72 to 31.72	−52.2
NRVD	286	188 to 431	360	281 to 470	25.6	<2		<2		
CMY and MYC	<5					<5				

IS: ischaemic stroke and haemorrhagic stroke; ICH: intracranial haemorrhage; SAH: subarachnoid haemorrhage; HHD: hypertensive heart disease; CMY and MYC: cardiomyopathy and myocarditis.

The overall death rate due to CVDs per 100 000 population showed a significant decrease from 402.75 (95% UI 361.2 - 443.37) in 1990 to 182.63 (95% UI 165.49 - 203.91) in 2017. In 2017, the age-standardized death rate due to the different CVDs showed a >50% reduction compared with the rates in 1990. The highest death rate reduction was observed for RHD, while the lowest reduction was observed in IHD (Table 1).

As shown in Table 2, the age-standardized rates of CVD mortality, all-cause mortality, mortality due to communicable diseases and mortality due to NCDs showed a significant decline in 2017 compared with 1990. However, the rate of change for CVDs and NCDs was lower compared with the rate of change for communicable, maternal, neonatal and nutritional diseases (Table 2).

**Table 2.** Comparisons of mortality reduction in all causes, communicable, maternal, nutritional and non-communicable diseases and CVDs between 2000 and 2017

	Deaths					The age-standardized death rate per 100 000				
	2000		2017			2000		2017		
Disease	n	95% UI	n	95% UI	Change, %	Rate	95% UI	Rate	95% UI	Change, %
All causes	937 863	907 341 to 969 789	536 990	511 092 to 566 670	−42.7	2061.3	1984.6 to 2138.9	960.8	916.8 to 1005.5	−53.4
Communicable, maternal, neonatal and nutritional diseases	659 189	631 342 to 687 894	298 486	276 467 to 322 759	−54.7	1098.3	1043.9 to 1157.1	372.63	346.7 to 412.8	−66.1
NCDs	180 427	167 648 to 196 607	196 669	182 158 to 209 046	9	772.9	723.25 to 819.9	518.7	478.89 to 551.43	−32.9
CVDs	63 283	58 507 to 68 364	58 719	53 177 to 65 228	−7.2	318.18	295.81 to 340.75	182.63	165.5 to 203.91	−42.6

### YLL and YLD

**Figure 2.**
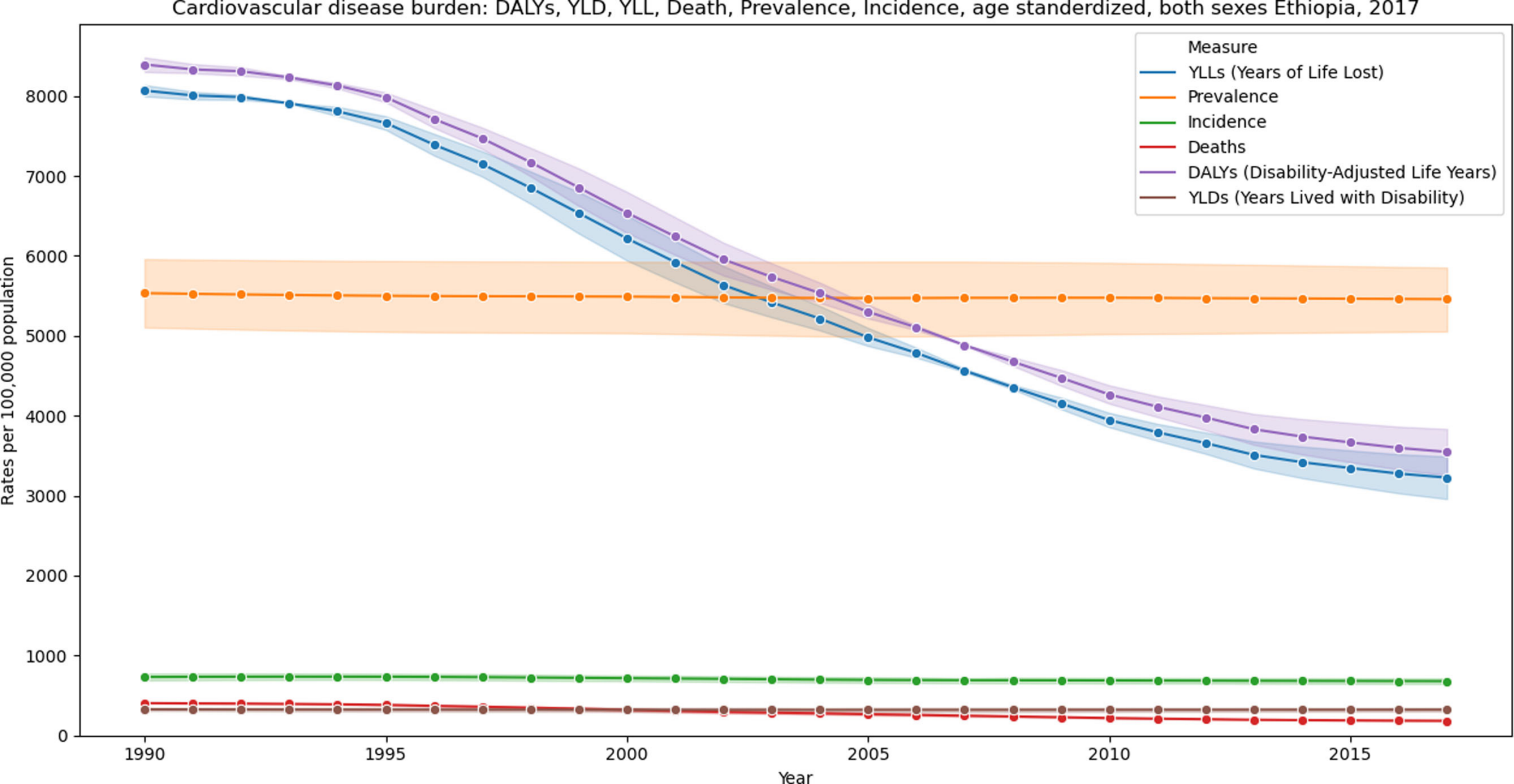
Trends in age-standardized death rate, DALYs, YLLs, YLDs and incidence and prevalence per 100 000 populations per year from 1990 to 2017, GBD data, Ethiopia.

The age-standardized CVD YLL decreased by 60%, from 8069.8 (95% UI 6979.56 - 9158.01) per 100 000 in 1990 to 3227.48 (95% UI 2925.39 - 3583.51) per 100 000 in 2017 (Figure 2). The age-standardized CVD YLD has remained stable over the years at 325.78 (95% UI 240.43 - 418.87) per 100 000 in 1990 and 322.07 (95% UI 235.93 - 414.65) per 100 000 in 2017 (Figure 2).

### DALYs

The age-standardized CVD DALYs have decreased by 57.7%, from 8395.6 (95% UI 7308.2 - 9499.8) in 1990 to 3549.6 (95% UI 3229.0 - 3911.9) in 2017 per 100 000 population (Figure 2). Cardiomyopathy has shown the lowest rate of decrease (25.2%) and intracranial haemorrhage showed the highest rate of decrease (65.7%) (Table 3).

**Table 3.** Total DALYs and age-standardized DALYs for component cardiovascular causes of death in 1990 and 2017, GBD data, Ethiopia

	DALYs					Age-standardized DALY rate per 100 000				
	1990		2017			1990		2017		
Disease	n	95% UI	n	95% UI	Change, %	Rate	95% UI	Rate	95%UI	Change, %
CVD	1 957 701.4	1 664 891.6 to 2 269 457.6	1 552 450.0	1 411 814.1 to 1 704 329.6	−20.7	8395.6	7308.2 to 9499.8	3549.6	3229.0 to 3911.9	−57.7
RHD	120 779.5	88 889.5 to 57 263.3	86 416.6	67 601.4 to 08 424.0	−28.4	300.2	231.1 to 284.2	105.6	82.4 to 131.3	−64.8
IHD	731 463.8	604 959.6 to 864 206.7	613 133.9	530 057.4 to 699 557.7	−16.2	3470.9	2951.0 to 3999.3	1521.7	1306.8 to 1747.5	−56.2
Stroke	704 182.9	578 419.5 to 867 207.1	496 015.4	431 630.2 to 563 763.3	−29.6	3180.6	2702.9 to 3801.2	1194.4	1026.4 to 1372.9	−62.4
IS	150 712.1	117 930.7 to 189 622.4	135 726.8	101 243.9 to 173 640.5	−9.9	843.9	673.4 to 1050.2	375.4	278.6 to 481.8	−55.5
ICH	492 156.1	378 735.8 to 650 316.5	313 740.5	272 517.5 to 358 931.3	−36.2	2135.3	1693.4 to 2738.1	733.3	626.5 to 852.4	−65.7
SAH	61 314.7	35 624.5 to 88 367.0	46 548.0	29 211.9 to 80 778.8	−24.1	201.4	117.7 to 301.0	85.6	52.3 to 157.8	−57.5
HHD	147 629.6	66 055.6 to 219 002.0	124 621.1	69 091.5 to 191 927.4	−15.6	747.3	369.2 to 1115.6	325.8	178.2 to 504.0	−56.4
NRVD	9393.9	5781.9 to 14 932.1	10 447.1	8634.5 to 12.851.8	11.2	39.8	27.2 to 57.7	22.8	18.2 to 28.9	−42.8
CMY and MYC	102 725.1	70 021.5 to 136 067.7	81 897.0	63 772.0 to 100 302.3	−20.3	204.3	148.8 to 271.9	111.9	83.8 to 137.4	−25.2

IS: ischaemic stroke and haemorrhagic stroke; ICH: intracranial haemorrhage; SAH: subarachnoid haemorrhage; HHD: hypertensive heart disease; CMY and MYC: cardiomyopathy and myocarditis.

### CVDs by age and sex

**Figure 3.**
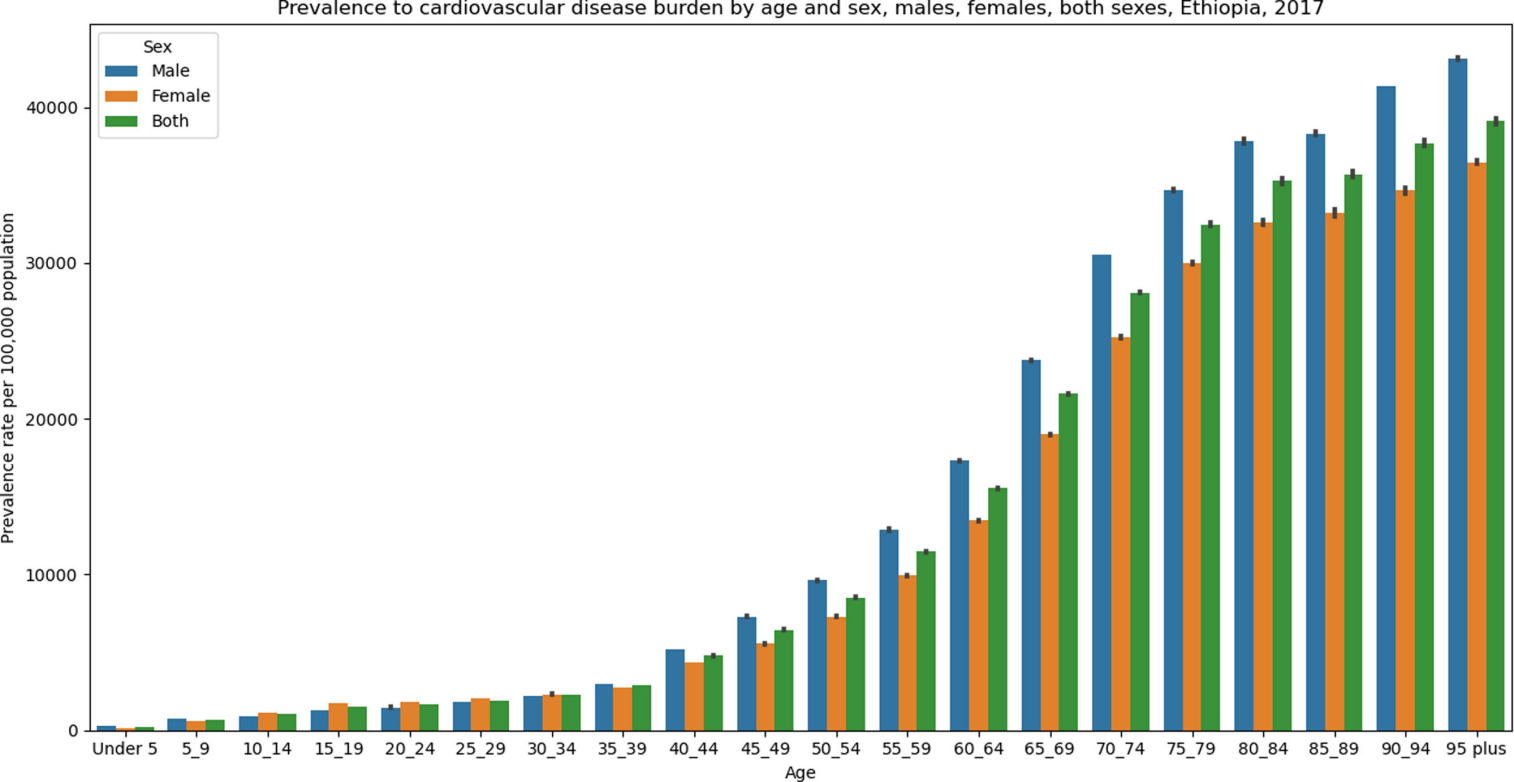
CVD prevalence rate by age groups and gender, GBD 2017 data, Ethiopia.

The GBD 2017 data show that the prevalence rate of CVDs has increased with age in Ethiopia (Figure 3). The increment is more pronounced at >35 y (Figure 3). There is no significant difference between males and females concerning age-standardized mortality and DALYs per 100 000 population in 2017. However, the age-standardized prevalence rate of CVD was significantly higher in males (5852.03 [95% UI 5606.46 - 6099.59]) than females (5053.57 [95% UI 4832.01 - 5278.25]) in 2017.

### Risk factors for CVD

**Figure 4.**
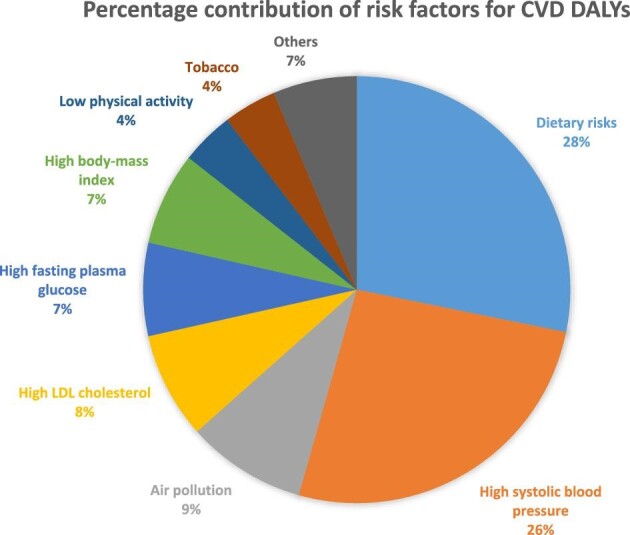
Number of DALYs for CVDs attributed to different risk factors in Ethiopia, GBD data, 2017.

In 2017 the leading risk factors for CVD in Ethiopia were dietary risk factors, high blood pressure and air pollution. Dietary risk factors accounted for 28% or 778 143.4 (95% UI 659 352.02 - 899 969.4) of CVD-caused DALYs, high blood pressure accounted for 26% or 701 299.6 (95% UI 605 764.3 - 799 216.4) of CVD-caused DALYs and air pollution (household air pollution and ambient particulate matter) accounted for 9% or 249 779.9 (95% UI 209 366.9 - 293 647.2) of CVD-caused DALYs (Figure 4).

## Discussion

The number of CVD cases in Ethiopia doubled in 2017 as compared with 1990. This might be explained by the observed high population growth coupled with an increased aging population caused by an increase in life expectancy. In Ethiopia, the life expectancy at birth has increased from 47.13 y in 1990 to 68.39 y in 2017.^19^

The age-standardized prevalence and incidence rate of CVD in Ethiopia has remained consistent throughout the last 28 y. The observed stable prevalence of CVDs despite the decrease in age-standardized mortality has not been the case for only Ethiopia. Similar patterns have been observed for global CVD, sub-Saharan Africa and eastern sub-Saharan Africa regional estimates.^19^

RHD, IHD and stroke were the leading prevalent CVDs across all age groups. When adjusted for age, IHD becomes the leading CVD followed by RHD and stroke. The age-adjusted prevalence rates of these leading CVDs were consistent over the past 28 y, irrespective of population growth and increasing life expectancy. Gender-specific analysis of the data indicates that RHD was more common among females than males, compared with IHD, which was more common among males.

There has been a rapid decrease in age-standardized all-cause mortality in the past 28 y in Ethiopia. This could be partly attributed to the decline in CVD mortality, particularly due to IHD and stroke, and a decrease in mortality due to communicable diseases and maternal and childhood diseases. In past decades, Ethiopia was known for the high burden of communicable, maternal, neonatal, child and nutritional diseases, where the majority of deaths in the country were attributed to these leading causes. Since the MDG era, Ethiopia has reported a significant reduction in the burden of communicable, maternal, neonatal, child and nutritional diseases. This uncovered the high burden of NCDs in the country and drew the focus of attention. According to the findings of the present study, in 1990 CVDs accounted for 7.6% of all causes of deaths, whereas in 2017 CVDs accounted for 10.9% of all causes of deaths. Ethiopia seems to be in an early phase of epidemiological transition due to rapid economic growth, urbanization and lifestyle change, where all are mentioned as reasons for the increase in NCDs as described in recent literature. Moreover, the observed high prevalence of RHD for the past 28 y might demonstrate the link between communicable and NCDs, as RHD is a sequela of some communicable diseases, which is commonly seen in the early phase of epidemiologic transition in many settings.^23–25^

The age-standardized CVD mortality rate decreased by 54.7% between 1990 and 2017, in contrast with the sub-Saharan regional average mortality estimate, which has shown a 1% increase.^26^ The data suggest the need for further investigation, as most of the sources of death data for the GBD are verbal autopsy and a limited number of surveys conducted in the country's capital city, Addis Ababa. Such data have a substantial risk of underestimating and/or miscategorizing the underlying causes of death in Ethiopia.

DALYs showed a 57% decrease from 1990 to 2017. Over the years, premature mortality consistently has been the greatest contributor to DALYs. This can be attributed to poor access to healthcare services, poor health service utilization and poor health-seeking behaviour of patients. Consequently most patients make hospital visits quite late. Furthermore, the overall healthcare service provision for CVDs in the country is underdeveloped.

According to the findings, IHD and stroke were the first and second leading GBD level 3 causes of CVD deaths in Ethiopia. Consistent with this finding, an epidemiological study that compiled data for the eastern sub-Saharan Africa region has reported stroke as the first and IHD as the second leading cause of death.^27^ The observed high death rate due to IHD and stroke in Ethiopia might be associated with recent urbanization, which could also be exacerbated by physical inactivity. According to a study published in 2019, 45% of the study population had low physical activity and only 37% reported high physical activity, while the national STEP wise approach to surveillance (STEPS) survey reported these figures as 26% and 42%, respectively.^28,29^ Additional factors including high saturated fat (palm oil) consumption,^30^ consumption of hydrogenated fatty acids and low consumption of fruits and/or vegetables (1.5%)^31^ might have contributed to the observed CVD-related deaths. Moreover, consumption of Omega 3 unsaturated fat, fruits and vegetables is reported to be low in Ethiopia.^31^ Our study showed that poor dietary habits and high systolic blood pressure were the main risk factors attributable to 54% of the DALYs (Figure 4). Age >35 y is also associated with a sharp increase in the prevalence of CVD in Ethiopia. This might be associated with the observed increase in life expectancy attributed to a significant decrease in mortality in children <5 y of age, which in turn increases the risk of CVD that occurs later in life proportionally.^19^

One of the limitations of this study is associated with the data source. The GBD has used different data sources to estimate the disease burden in Ethiopia. Most of these data sources were on communicable diseases and maternal, neonatal, child and nutritional diseases. The data source for CVDs was limited to the population censuses and to health and demographic surveys conducted in Ethiopia as opposed to the more reliable and diverse sources of CVD data in resource-rich settings. In this GBD 2017 result, death due to CVD was estimated mainly using Addis Ababa mortality surveillance data and two other published reports on the causes of death. Both of these used verbal autopsy to verify the death and causes of death. Therefore most of the estimates might not be precise. Despite this, this article presents the burden of CVD as the first national estimate for Ethiopia and underlines the need to establish a national system to estimate national burden and causes of death due to CVD. The findings from this article will be valuable policy makers and can serve as a baseline for prospective national CVD control efforts in Ethiopia.

## Conclusions

Ethiopia has a high burden of CVD, but it was masked by the high burden of communicable diseases. A recent achievement in the control of communicable, maternal and child health-related conditions in Ethiopia has showcased the increasing burden of CVD. Hence it is timely to consider CVD as one of the disease priorities of the country and to strengthen the people-centred health system that enables prevention and treatment. Furthermore, we recommend the FMoH and health development partners conduct a national survey to better estimate the prevalence of CVD in Ethiopia.

## References

[1] World Health Organization . Cardiovascular diseases (CVDs). Available from: https://www.who.int/en/news-room/fact-sheets/detail/cardiovascular-diseases-(cvds) [accessed 21 February 2019].

[2] World Health Organization . Cardiovascular diseases. Available from: https://www.who.int/cardiovascular_diseases/en/ [accessed 21 February 2019].

[3] World Health Organization . Global health estimates 2016 summary tables: YLDs by cause, age, and sex, by World Bank income group, 2000–2016. Geneva: World Health Organization; 2018. Available from: https://www.who.int/healthinfo/global_burden_disease/GHE2016_YLD_WBI_2000_2016_.xls [accessed 21 February 2019].

[4] Tekola-Ayele F , Adeyemo AA, Rotimi CN. Genetic epidemiology of type 2 diabetes and cardiovascular diseases in Africa. Prog Cardiovasc Dis. 2013;56(3): 10.1016/j.pcad.2013.09.013. 10.1016/j.pcad.2013.09.013 PMC3840391 24267432

[5] Misganaw A , Haregu TN, Deribe K et al. National mortality burden due to communicable, non-communicable, and other diseases in Ethiopia, 1990–2015: findings from the Global Burden of Disease Study 2015. Popul Health Metr. 2017;15:29. 28736507 10.1186/s12963-017-0145-1 PMC5521057

[6] Ethiopia 2017 voluntary national review on SDGs: government commitments, national ownership and performance trends. Addis Ababa: National Planning Commission; 2017, p. 21–22. Available from: http://www.et.undp.org/content/dam/ethiopia/docs/2017/The%202017%20VNRs%20on%20SDGs_Ethiopia%20(Eng)%20Web%20version%20.pdf [accessed 21 February 2019].

[7] Blahos J , Kubastova B. The survey of 11,170 patient treated in the Ras Mekonnen Hospital in Harar. Ethiop Med J. 1963;1:190.

[8] World Bank . The World Bank in Ethiopia. Overview. Available from: https://www.worldbank.org/en/country/ethiopia/overview [accessed July 2020].

[9] World Bank Group . Ethiopia urbanization review: urban institutions for a middle-income Ethiopia. Washington, DC: World Bank; 2015. Available from: https://openknowledge.worldbank.org/handle/10986/22979.

[10] Teklu B , Parry EH, Pavlica D. Ethiopian cardiovascular studies. X. Normal variations of the electrocardiogram in Ethiopians. Ethiop Med J. 1971;9(3):133–9. 5150530

[11] Misganaw A , Mariam DH, Araya T. The double mortality burden among adults in Addis Ababa, Ethiopia, 2006–2009. Prev Chronic Dis. 2012;9:E84. 22498035 10.5888/pcd9.110142 PMC3396553

[12] Fantahun M , Degu G. Burden of diseases in Amhara region, Ethiopia. Ethiop Med J. 2004;42(3):165–72. 16895033

[13] Federal Democratic Republic of Ethiopia, Ministry of Health . National strategic action plan (NSAP) for prevention & control of non-communicable diseases in Ethiopia 2014–2016. Available from: https://www.iccp-portal.org/system/files/plans/ETH_B3_National%20Strategic%20Action%20Plan%20%28NSAP%29%20for%20Prevention%20and%20Control%20of%20Non-Communicable%20Diseases%20-%20Final.pdf [accessed June 2020].

[14] Ethiopian Public Health Institute . Ethiopia STEPS report on risk factors for chronic non communicable diseases and prevalence of selected NCDs. Available from: https://www.who.int/ncds/surveillance/steps/Ethiopia_2015_STEPS_Report.pdf [accessed June 2020].

[15] Argaw S , Tesfahun E, Dereseh BT et al. Determinants of selected cardiovascular diseases among adult patients at cardiac clinic of Debre Berhan Referral Hospital, Ethiopia: unmatched case–control study. Cardiovasc Ther. 2020;2020:7036151. 10.1155/2020/7036151 PMC7273416 32547636

[16] Institute for Health Metrics and Evaluation . GBD Frequently asked questions. Available from: http://www.healthdata.org/gbd/faq#What%20is%20GBD%202010%20and%20why%20is%20it%20important? [accessed September 2019].

[17] GBD 2017 DALYs and HALE Collaborators . Global, regional, and national disability-adjusted life-years (DALYs) for 359 diseases and injuries and healthy life expectancy (HALE) for 195 countries and territories, 1990–2017: a systematic analysis for the Global Burden of Disease Study 2017. Lancet. 2018;392(10159):1859–1922. 10.1016/S0140-6736(18)32335-3 PMC6252083 30415748

[18] GBD 2017 Causes of Death Collaborators . Global, regional, and national age-sex-specific mortality for 282 causes of death in 195 countries and territories, 1980–2017: a systematic analysis for the Global Burden of Disease Study 2017. Lancet. 2018;392(10159):1736–88. 30496103 10.1016/S0140-6736(18)32203-7 PMC6227606

[19] GBD 2017 Disease and Injury Incidence and Prevalence Collaborators . Global, regional, and national incidence, prevalence, and years lived with disability for 354 diseases and injuries for 195 countries and territories, 1990–2017: a systematic analysis for the Global Burden of Disease Study 2017. Lancet. 2018;392(10159):1789–1858. 30496104 10.1016/S0140-6736(18)32279-7 PMC6227754

[20] GBD 2017 Mortality Collaborators . Global, regional, and national age-sex-specific mortality and life expectancy, 1950–2017: a systematic analysis for the Global Burden of Disease Study 2017. Lancet. 2018;392(10159):1684–735. 30496102 10.1016/S0140-6736(18)31891-9 PMC6227504

[21] GBD 2017 Population and Fertility Collaborators . Population and fertility by age and sex for 195 countries and territories, 1950–2017: a systematic analysis for the Global Burden of Disease Study 2017. Lancet. 2018;392(10159):1995–2051. 30496106 10.1016/S0140-6736(18)32278-5 PMC6227915

[22] GBD 2017 Risk Factor Collaborators . Global, regional, and national comparative risk assessment of 84 behavioral, environmental and occupational, and metabolic risks or clusters of risks for 195 countries and territories, 1990–2017: a systematic analysis for the Global Burden of Disease Study 2017. Lancet. 2018;392(10159):1923–94. 30496105 10.1016/S0140-6736(18)32225-6 PMC6227755

[23] Lulu K , Berhane Y. The use of simplified verbal autopsy in identifying causes of adult death in a predominantly rural population in Ethiopia. BMC Public Health. 2005;5:58. 15935096 10.1186/1471-2458-5-58 PMC1164421

[24] Weldearegawi B , Ashebir Y, Gebeye E et al. Emerging chronic non-communicable diseases in rural communities of Northern Ethiopia: evidence using population-based verbal autopsy method in Kilite Awlaelo surveillance site. Health Policy Plan. 2013;28(8):891–8. 23293101 10.1093/heapol/czs135

[25] Anteneh A , Araya T, Misganaw A. Factors associated with place of death in Addis Ababa, Ethiopia. BMC Palliat Care. 2013;12:14. 23530478 10.1186/1472-684X-12-14 PMC3616966

[26] Mensah GA , Roth GA, Sampson UK et al. Mortality from cardiovascular diseases in sub-Saharan Africa, 1990–2013: a systematic analysis of data from the Global Burden of Disease Study 2013. Cardiovasc J Afr. 2015;26(2 Suppl 1):S6–10. 25962950 10.5830/CVJA-2015-036 PMC4557490

[27] Moran A , Forouzanfar M, Sampson U et al. The epidemiology of cardiovascular diseases in sub-Saharan Africa: the Global Burden of Diseases, Injuries and Risk Factors 2010 Study. Prog Cardiovasc Dis. 2013;56(3):234–9. 24267430 10.1016/j.pcad.2013.09.019 PMC4031901

[28] Mengesha MM , Roba HS, Ayele BH et al. Level of physical activity among urban adults and the socio-demographic correlates: a population-based cross-sectional study using the global physical activity questionnaire. BMC Public Health. 2019;19:1160. 31438909 10.1186/s12889-019-7465-y PMC6704679

[29] World Health Organization . Ethiopia (Addis Ababa) STEPS Survey 2006 fact sheet. Available from: https://www.who.int/ncds/surveillance/steps/2006_Ethiopia_FactSheet_EN.pdf [accessed 8 September 2020].

[30] Habte K , Kebede A, Tessema M et al. Safety and nutritional quality of commonly consumed commercial edible oils in Addis Ababa. Ethiop J Public Health Nutr. 2017;1(2):72–7.

[31] Gelibo T , Amenu K, Tadele T et al. Low fruit and vegetable intake and its associated factors in Ethiopia: a community based cross sectional NCD steps survey. Ethiop J Health Dev. 2017;31(Special Issue):355–61.

